# Gelatinase B/MMP-9 in Tumour Pathogenesis and Progression

**DOI:** 10.3390/cancers6010240

**Published:** 2014-01-27

**Authors:** Antonietta Rosella Farina, Andrew Reay Mackay

**Affiliations:** Department of Applied Clinical and Biotechnological Sciences, University of L’Aquila, Via Vetoio, Coppito 2, L’Aquila 67100, Italy; E-Mail: antonietta.farina@univaq.it

**Keywords:** gelatinase B/MMP-9, tumour progression, angiogenesis, metastasis, inflammation, epithelial-mesenchymal transition, invasion, motility, immune surveillance, gelatinase B/MMP-9 inhibitors

## Abstract

Since its original identification as a leukocyte gelatinase/type V collagenase and tumour type IV collagenase, gelatinase B/matrix metalloproteinase (MMP)-9 is now recognised as playing a central role in many aspects of tumour progression. In this review, we relate current concepts concerning the many ways in which gelatinase B/MMP-9 influences tumour biology. Following a brief outline of the gelatinase B/MMP-9 gene and protein, we analyse the role(s) of gelatinase B/MMP-9 in different phases of the tumorigenic process, and compare the importance of gelatinase B/MMP-9 source in the carcinogenic process. What becomes apparent is the importance of inflammatory cell-derived gelatinase B/MMP-9 in tumour promotion, early progression and triggering of the “angiogenic switch”, the integral relationship between inflammatory, stromal and tumour components with respect to gelatinase B/MMP-9 production and activation, and the fundamental role for gelatinase B/MMP-9 in the formation and maintenance of tumour stem cell and metastatic niches. It is also apparent that gelatinase B/MMP-9 plays important tumour suppressing functions, producing endogenous angiogenesis inhibitors, promoting inflammatory anti-tumour activity, and inducing apoptosis. The fundamental roles of gelatinase B/MMP-9 in cancer biology underpins the need for specific therapeutic inhibitors of gelatinase B/MMP-9 function, the use of which must take into account and substitute for tumour-suppressing gelatinase B/MMP-9 activity and also limit inhibition of physiological gelatinase B/MMP-9 function.

## 1. Introduction

Since the original identification of galetinase B/matrix metalloproteinase (MMP)-9, as a human leukocyte gelatinase [1,2,3,4], its characterisation as a type V collagenase [5], the observation that malignant tumour cells express an identical enzyme that associates with metastatic behaviour and degrades type IV collagen under certain conditions [6,7,8,9,10] and its subsequent cloning from HT-1080 fibrosarcoma cells [11], research into the physiological and pathological functions of gelatinase B/MMP-9, in contrast to almost all other MMPs, has continued to increase at a steady rate [12,13]. Gelatinase B/MMP-9 involvement in malignant tumour progression has now moved on from original concepts of an almost exclusive role in matrix degradation, associated with tumour invasion, to include roles in almost all aspects of tumour biology, ranging from initiation and early progression, to angiogenesis, dissemination, invasion and motility, formation of the cancer stem cell niche, regulation of tumour immunological surveillance, metastatic site preparation and promotion of metastatic growth.

In the present article, following a briefly description of the human gelatinase B/MMP-9 gene, protein and mechanisms that regulate its expression, activation and activity, we review current concepts concerning gelatinase B/MMP-9 involvement in tumour progression, starting with the genetic damage that results in transformation and accompanies tumorigenicity, neoplastic expansion and the accumulation of mutations, increased survival, tumour-associated angiogenesis, adhesive interactions, matrix degradation and the loss of basement membrane, tumour cell invasion, motility, intravasation and extravasation, evasion of immunological surveillance, and regulation of the cancer stem cell and metastatic niches. We also review tumour-associated mechanisms that alter the equilibrium between gelatinase B/MMP-9 and its inhibitors and address novel ways to inhibit gelatinase B/MMP-9 involvement in tumour progression.

## 2. The Gelatinase B/MMP-9 Gene and mRNA

The human gelatinase B/MMP-9 gene localises to chromosome 20q11.2-q13.1, consists of 7,654 bases, starting from 44,637,547 bp from pter to 44,645,200 bp from pter, and is arranged into 13 exons [14]. The 2.2 kb gelatinase B/MMP-9 promoter resembles that of MMP-1 and MMP-3 rather than the MMP-2 one, and contains a TATA motif at position -29, GC box at position -563, TGF-beta inhibitory element at position -474, AP-1 elements at positions -79 and -209, three Ets binding PEA3 sites between -599 and -531, an NF-κB element at positions -600 and -328, two AP-2 elements and a (CA)n segment [12]. Gelatinase B/MMP-9 is transcribed as a single 2.4 kb mRNA species and alternative splice variants have not been reported [15,16]. The gelatinase B/MMP-9 3'-UTR contains functional bindings sites for miR-491-5p, miR-885-5p [17] and miR-211 [18].

### Gelatinase B/MMP-9 SNPs

A single C > T nucleotide polymorphism at position −1562 within the gelatinase B/MMP-9 promoter, originally associated with coronary atherosclerosis [19], deregulates gelatinase B/MMP-9 expression and associates with gastric tumour progression [20], susceptibility to oral squamous cell carcinoma [21,22,23], nasopharyngeal carcinoma [24], squamous cell carcinoma of the lung [25] and oesophageal squamous cell carcinoma [26], and also associates with a higher risk of metastasis in the Asian, but not the European population [27]. Polymorphisms in the length of (CA)n sequence within the gelatinase B/MMP-9 promoter have been reported, with lengths of (CA)21 and (CA)23 shown to increase gelatinase B/MMP-9 transcription [28,29,30]. This region is close to TRE, SP1 and NF-κB cis elements and may alter their function. The (CA)n element binds a specific DNA binding protein, dependent upon CA number [30]. The gelatinase B/MMP-9 polymorphism Rs1056628CC, detected within the Chinese population, is characterised by a change in base 2182 from A to C within the 3'-UTR miR491-5p binding sequence and increases gelatinase B/MMP-9 expression, potentially through altered miR-491-5p binding [31]. Two gelatinase B/MMP-9 coding region single nucleotide polymorphisms rs2250889 (P574R) and rs17576 (R279Q) have been associated with risk of lung cancer and lung cancer metastasis [32], and with reduced overall survival of patients with loco regionally advanced nasopharyngeal carcinoma, characterised by increased tissue gelatinase B/MMP-9 expression [33,34], lymph node metastasis in gastric cancer [35] and risk of gallbladder cancer [36] but these SNPs do not appear to associate with colon cancer susceptibility in a Chinese cohort study [37]. In addition to these reports, gelatinase B/MMP-9 coding region SNPs Arg279Gln and Arg668Gly may represent potential predictors of survival in Chinese patients with non-small cell lung cancer [38].

## 3. The Gelatinase B/MMP-9 Protein

We direct the reader to the excellent and extensive reviews by Van den Steen and colleagues, and Vandooren and colleagues [12,13], concerning gelatinase B/MMP-9 biochemistry and molecular biology. Briefly, the gelatinase B/MMP-9 protein is a multi-domain metallo-enzyme, with a catalytic site composed of a metal binding domain separated from the active site by three fibronectin repeats that facilitate the degradation of large substrates such as elastin and denatured collagens. Within this region the amino acids Asp309, Asn319, Asp232, Tyr320 and Arg3076 are important for gelatin binding. The catalytic site is maintained inactive by an amino-terminal pro-peptide PRCGXPD, with the cysteine coordinated with the catalytic Zn^2+^. The COOH terminus of gelatinase B/MMP-9 contains a hemopexin domain that regulates substrate binding, interacts with inhibitors and facilitates cell surface binding. A central O-glycosylated domain provides molecular flexibility, regulates gelatinase B/MMP-9 substrate specificity, gelatinase B/MMP-9-dependent invasion, interaction with TIMP and cell surface localisation. This domain facilitates the movement of gelatinase B/MMP-9 along macromolecular substrates and unwinds collagen initially cleaved by other enzymes, permitting gelatinase B/MMP-9-mediated degradation [12,13].

### 3.1. Gelatinase B/MMP-9 Catalytic Site

Within the gelatinase B/MMP-9 catalytic domain the amino acid Glu402 and Zn^2+^ ion are essential for function, amino acids Leu397 and Ala406 are important for general catalytic activity, Asp410 enhances type V collagenolytic activity, Pro415 enhances gelatinolytic activity [39] and Gly substitution of Glu415 renders gelatinase B/MMP-9 collagenolytic [40]. The propeptide domain contains a “cysteine switch” sequence that binds to the catalytic Zn^2+^ ion, inhibiting catalytic activity. Gelatinase B/MMP-9 activation is achieved by proteolytic removal of this sequence by enzymes that include: trypsin, cathepsin G, kallikrien, elastase, chymase, neutrophil elastase and the MMPs-1, -2, -3, -7, -10, -13 and -26 [12]. Debate exists, however, as to whether plasmin can directly activate gelatinase B/MMP-9 [12,41]. Indirect plasmin-mediated gelatinase B/MMP-9 activation is achieved via MMP-1, MMP-3 and MMP-7 [12]. In addition to proteolytic gelatinase B/MMP-9 activation, agents that modify the interaction between the pro-peptide cysteine and the catalytic site Zn^2+^ ion, such as ionic detergents, organo-mercurials, oxidising agents, S-nitrosylation and S-glutothiolation can also activate gelatinase B/MMP-9 [12,42,43,44]. The gelatinase B/MMP-9 catalytic domain contains six disulphide bonds that are necessary for intracellular trafficking and gelatinase B/MMP-9 secretion [45]. The gelatinase B/MMP-9 catalytic site also contains cryptic plasmin degradation sites that are exposed by divalent cation chelators and by the bisphosphonate alendronate (Fosamax) and upon degradation irreversibly inhibit gelatinase B/MMP-9 catalytic activity [41].

### 3.2. Gelatinase B/MMP-9 Hemopexin Domain

The gelatinase B/MMP-9 hemopexin domain exhibits a relatively unique covalent structure in which Cys516 and Cys704 form a disulphide bridge, which is involved in domain function but is not required for gelatinase B/MMP-9 secretion [45,46]. This domain facilitates interactions with substrates, gelatinase B/MMP-9 oligomerisation, binds the carboxyl terminal of TIMP-1, binds cell surface proteins such Ku70/80 and LRP, and upon binding appropriate substances, such as heme, also mediates autocatalytic gelatinase B/MMP-9 activation [47]. Divergent disulphide bridging between the 17-cysteine residues within gelatinase B/MMP-9 regulates structure and function. Disulphide bridging within fibronectin repeats are essential for gelatinase B/MMP-9 secretion. Hemopexin domain function depends upon disulphide bridging and disulphide bridging between the *O*-glycosylation or hemopexin domains facilitates gelatinase B/MMP-9 dimerization or oligomerisation, promoting CD44 binding, which results in activation of the EGF receptor and subsequent ERK/1/2 mediated cancer cell migration [46,48]. Gelatinase B/MMP-9 hemopexin domain hetero-dimerization with proteins such as TIMP-1 and NGAL protects gelatinase B/MMP-9 against proteolytic degradation.

### 3.3. Gelatinase B/MMP-9 O-Glycosylation Domain

The O-glycosylated domain of gelatinase B/MMP-9, also known as the type V collagen-like domain, represents a 64 amino acid linker containing 22 proline residues, six glycine residues and approximately 12–14 *O*-linked glycans [49]. This domain is active in hemopexin domain orientation, which is important for molecular interactions with exogenous proteins, including gelatinase B/MMP-9 substrates [49]. The removal of this domain reduces gelatinase B/MMP-9 specificity for macromolecular substrates, including gelatin [13].

### 3.4. Truncated Gelatinase B/MMP-9 Isoforms

Several truncated gelatinase B/MMP-9 isoforms have been described that include proteolytically active fragments derived from autocatalysis and exogenous proteolytic degradation. The 65 kDa gelatinase B/MMP-9 catalytically active fragment generated by MMP-3 is deleted of COOH terminal sequence and escapes TIMP-1 inhibition. KLK7 and meprin-α also remove this domain from gelatinase B/MMP-9 [50,51,52]. A novel 82 kDa inactive pro-gelatinase B/MMP-9 form has been described in human leukaemic cells, which also escapes TIMP inhibition [53,54] and a similar sized human pro-gelatinase B/MMP-9 isoform is generated by the action of plasmin [41].

## 4. Gelatinase B/MMP-9 Substrates

Gelatinase B/MMP-9 was originally characterised as a gelatinase/V collagenase [1,2,3,4,5], and was later attributed type IV collagenolytic activity [6,11]. Although there is controversy surrounding the susceptibility [55] or resistance [8,40,56] of triple helical domains of collagens to degradation by gelatinase B/MMP-9, the capacity of gelatinase B/MMP-9 to degrade native type IV collagen may be limited, therefore, to non-triple helical, less-disulphide cross-linked or pre-digested molecular forms of type IV collagen [8,57,58]. It remains debatable whether activated gelatinases alone degrade type IV collagen within the context of an insoluble basement membrane [8,57,58,59]. Gelatinase B/MMP-9 does, however, degrade basement membrane laminin, disrupting basement membrane structure, tissue architecture [60] and inducing apoptosis [61]. In addition to its capacity to degrade extracellular matrix components, recent reports have characterized an ever-increasing array of substrates susceptible to degradation by gelatinase B/MMP-9, dramatically widening the potential physiological and pathological sphere of gelatinase B/MMP-9 influence. Gelatinase B/MMP-9 exhibits substrate specificity for cytokines, chemokines and growth factors within the extracellular compartment and may also degrade nuclear, mitochondrial and cytoplasmic substrates (Table 1). For a broad spectrum of gelatinase B/MMP-9 substrates, both old and new, we direct reader to the following articles [52,55,56,57,58,59,60,61,62,63,64,65,66,67,68,69,70,71,72,73,74,75,76,77,78,79,80,81,82,83,84,85,86,87,88,89,90,91,92,93,94,95,96,97,98,99,100,101,102,103,104,105,106,107,108,109,110,111,112,113,114,115,116,117,118]. 

**Table 1:** Update of Gelatinase B/MMP-9 substrates. Substrates (sub) and MMP-9 origins are provided: human (hu), mouse (mu), bovine (bo) and rabbit (ra).

Class	Substrate	Substrate/MMP-9 source	[Refs]
**ECM Substrates**	Collagen type I	(bo/mu sub/hu MMP-9)	[55]
	Collagen type II	(hu sub/MMP-9)	[56]
	Collagen III	(bo sub/hu MMP-9)	[55]
	Collagen IV	(hu/mu sub/MMP-9)	[8,57,58,66,67,68,69,70]
	Collagen V	(hu sub/MMP-9)	[4,8,68]
	Collagen VI	(hu sub/MMP-9)	[70]
	Collagen α1 and α2 (VI)	(hu sub/MMP-9)	[62]
	Collagen α1 (XI)	(hu sub/MMP-9)	[71]
	Collagen α1 (XVIII)	(hu sub/MMP-9)	[70,72]
	Procollgen lysine-2-oxygluterate-5 dioxygenase-1	(hu sub/MMP-9)	[62]
	Periostin	(hu sub/MMP-9)	[70]
	Galectin-1	(hu sub/MMP-9)	[62,65]
	Galectin 3	(hu sub/MMP-9)	[73]
	Fibronectin	(hu sub/MMP-9)	[68,70,74]
	Laminin	(mu sub/hu MMP-9)	[60,62,68]
	Tenascin C	(hu sub/MMP-9)	[70,74,75]
	Tenascin X	(hu sub/MMP-9)	[70]
	Thrombospondin-2	(hu sub/MMP-9)	[65]
	Insulin growth factor binding protein 4	(hu sub/MMP-9)	[65]
	Cystatin C	(hu sub/MMP-9)	[65]
	Elastin	(Bo/mu sub/hu/mu MMP-9)	[76,77]
	Vitronectin	(hu sub/MMP-9)	[78]
	Entactin	(mu sub/hu MMP-9)	[79]
	Heparan sulphate	(hu sub/MMP-9)	[80]
**Cell surface substrates**	ICAM-1	(hu sub/MMP-9)	[81,82]
	uPAR	(hu sub/MMP-9)	[83]
	Laminin receptor	(Xenopus sub/hu MMP-9)	[84]
	IL2Rα	(hu sub/MMP-9)	[85,86]
	proTNFα	(hu sub/MMP-9)	[87]
	IL-1β	(hu sub/MMP-9)	[88,89]
	Kit ligand	(mu/hu sub/MMP-9)	[90,91]
	β2 integrin subunit	(mu sub/MMP-9)	[92]
	proTGFβ	(mu sub/MMP-9)	[93]
	HB-EGF	(hu sub/MMP-9)	[94]
	Occludin tight junction protein	(bo sub/huMMP-9)	[95]
	Syndecan 1 and 4	(mu sub/MMP-9)	[96,97]
	Serpin α-1 proteinase inhibitor	(mu sub/MMP-9)	[98]
	myelin basic protein	(hu sub/MMP-9)	[99]
	NG2 Proteoglycan	(hu sub/MMP-9)	[100]
	β-distroglycan	(mouse substrate/MMP-9 ?)	[101]
	Soluble beta amyloid protein	(hu sub/MMP-9)	[102,103]
	Fibrilar beta amyloid protein	(mu sub/MMP-9 ?)	[103]
	ADAMTS-4 (aggrecanase-1)	(hu sub/MMP-9)	[104]
**Candidate cell surface substrates**	Angiopoetin 1 receptor Tie2	(hu sub/MMP-9)	[62]
	Neuropilin 1	(hu sub/MMP-9)	[62]
	Integrin α3	(hu sub/MMP-9)	[62]
	Clatherin heavy chain CLH17	(hu sub/MMP-9)	[62]
	CD166/ALCAM	(hu sub/MMP-9)	[62]
	Saposin A	(hu sub/MMP-9)	[62]
	Semaphorin 7A	(hu sub/MMP-9)	[62]
**CC Chemokines**	CCL7	(mu sub/MMP-9)	[105]
	CCL11 (Eotaxin)	(mu sub/MMP-9)	[105]
	CCL17 (TARC)	(mu sub/MMP-9)	[105]
**CXC Chemokines**	CXCL1/NAP-3	(hu sub/MMP-9)	[106]
	CXCL4/PF4	(hu sub/MMP-9)	[106]
	CXCL8/IL-8	(hu sub/MMP-9)	[106]
	CXCL7/CTAP-III	(hu sub/MMP-9)	[106]
	CXCL9/ MIG	(hu sub/MMP-9)	[107]
	CXCL10/IP-10	(hu sub/MMP-9)	[107]
	CXCL6/GCP-2	(hu/mu sub/hu MMP-9)	[107]
	CXCL5/ENA78	(hu sub/MMP-9)	[107]
	CXCL11/ITAC	(hu sub/MMP-9)	[108]
	CXCL12/SDF-1	(hu sub/MMP-9)	[109]
**Other Substrates**	Leukaemia inhibitory factor (LIF)	(hu sub/MMP-9)	[62]
	Protease nexin-1	(hu sub/MMP-9)	[62]
	Granulins precursor acrogranin	(hu sub/MMP-9)	[62]
	Hsp90	(hu sub/MMP-9)	[62]
	uPA precursor	(hu sub/MMP-9)	[62]
	tPA precursor	(hu sub/MMP-9)	[62]
	C1q	(hu sub/MMP-9)	[110]
	C1r-A	(mu sub/hu MMP-9)	[65]
	Pyruvate kinase isoenzymes M1/M2	(ra sub/hu MMP-9)	[65]
	Collagenase 3 (MMP-13)	(hu sub/MMP-9)	[62]
	Dickkopf-1	(hu sub/MMP-9)	[111]
	Dickkopf-3 tumour suppressor	(hu sub/MMP-9)	[65]
	DJ-1 oncogene	(hu sub/MMP-9)	[111]
	Follistain-like 3	(hu sub/MMP-9)	[111]
	Neuron specific enolase	(hu sub/MMP-9)	[111]
	Nieman-Pick C2	(hu sub/MMP-9)	[111]
	Proglanulins	(hu sub/MMP-9)	[111]
	Ym 1	(mu sub/MMP-9)	[105]
	S100A8 proinflammatory protein	(mu sub/MMP-9)	[105]
	S100A9 proinflammatory protein	(mu sub/MMP-9)	[105]
	Plasminogen	(hu sub/MMP-9)	[112,113]
	Mature NGF	(mu sub/MMP-9)	[114]
	Interferon-β	(hu sub/MMP-9)	[115]
	KISS-1 metastasis suppressor	(hu sub/MMP-9)	[116]
	Tau	(hu sub/MMP-9)	[117]
	VEGF	(mu sub/MMP-9 not hu MMP-9)	[80,118]

## 5. Gelatinase B/MMP-9 Transcription and Translation

**Figure 1:**
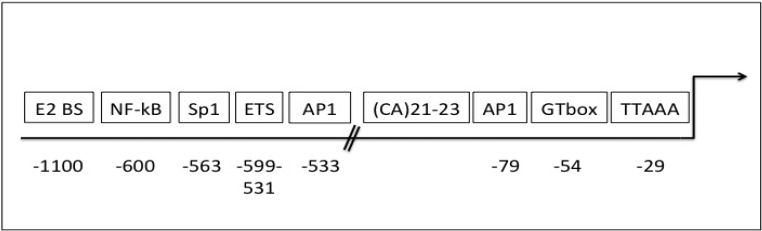
Localisation of functional transcriptional elements within the human MMP-9 promoter, displaying the positions, relative to the MMP-9 translational start site, for E2 protein (E2 BS), nuclear factor-kappa binding (NF-κB), specific protein-1 (Sp1), E26 transformation specific (ETS), CA repeat, activator protein-1 (AP1), GTbox and Tata box binding sites.

The 2.2 kb human *gelatinase B/MMP-9* promoter contains a TATA-like motif at position −29 but no CAAT-like motif. Relative to the transcriptional start site, functional transcription sites include: an SP1 binding GC box located at −563, a retinoblastoma binding element or GT box that also binds SP1 at position −54, and three additional GT boxes. In addition to a TGF-β1 inhibitory element at −474 bp and 4 potential AP-1 binding elements, the functional AP-1 site at position −79 is essential for basal and jun/Fos induced expression in HT-1080 and osteosarcoma cells [119], three functional PEA3/Ets binding sites localise between −599 and −531 are also involved in basal gelatinase B/MMP-9 transcription [119,120]. A functional NF-κB binding site is located at −600 and a second site at −328 bp [121], and potentially functional inhibitory AP-2-like binding sites immediately upstream of the GC-box that interferes with Sp-1 binding [122], an alternating microsatellite CA sequence in close proximity to the AP1 site at position −79 [12] (Figure 1).

Synergism between transcriptional elements characterises basal-, cytokine- and phorbol ester-induced gelatinase B/MMP-9 transcription, with the AP-1 element at position −79 necessary, but not sufficient for transcription, cooperating with NF-κB (−600) and SP1 (−563) elements, respectively [119]. The NF-κB element (−600) is required for gelatinase B/MMP-9 transcription induced during spontaneous epithelial to neuroblast transition and by all-*trans*-retinoic acid in human neuroblastoma cells [123,124], by TNF-α in HT-1080 fibrosarcoma cells and rabbit fibroblasts [119,125,126], IL1β [127], Bcl2 [128], HIV-1-Tat [129], KiSS1 [130], synergistic combinations of cytokines and growth factors [126,131] and thioredoxin [132], acting in concert with other elements including the AP-1 site at position −79. The Ets element at −541 acting together with the AP-1 element at position −533 regulates gelatinase B/MMP-9 transcription induced by c-Ha- Ras, EGF and fibroblast cell contact [12,120,133]. Both RBE (−54) and AP-1 (−79) elements regulate v-Src induced gelatinase B/MMP-9 transcription in fibrosarcoma cells, c-Ha-Ras induced gelatinase B/MMP-9 transcription in adenocarcinoma cells and c-Ha-ras/v-myc-induced gelatinase B/MMP-9 transcription in rat embryo cells [133]. The RBE element (−54) also functions together with the NF-κB element (−600) in gelatinase B/MMP-9 transcription induced by spontaneous epithelial to neuroblast conversion exhibited by SK-N-SH neuroblastoma cells [123]. In general, gelatinase B/MMP-9 transcription with few exceptions depends upon the concerted interaction between several transcriptional cis elements and cognate transcription factors, with particular important roles highlighted for AP1 (−79) and NF-κB (−600) elements, with NF-κB and SP1 transcription factors specific determinant for gelatinase B/MMP-9 expression. Recently, a functional binding site for the E2 protein expressed by human oncogenic papilloma virus 8 has been characterised at position −1100 of the human MMP-9 promoter and shown to promote MMP-9 transcription [134].

The gelatinase B/MMP-9 protein is constitutively expressed by only a limited number of cell types, such as keratinocytes, macrophages, polymorphonuclear leukocytes and some malignant tumour cell lines, including MDA-MB-231 breast cancer, HT-1080 fibrosarcoma and A2058 melanoma cell lines [12,15] and is readily induced in wide range of normal and tumour cell types by pro-inflammatory cytokines, activators of PKC and growth factors with gelatinase B/MMP-9 expression regulated through inhibitory STAT and SMAD pathways and stimulatory PKC, Ras/MAPK, TRAD/TRAF, MEK/JNK, ASK/MKK and IRAK/TRAF pathways [12,13,15].

Gelatinase B/MMP-9 expression is also regulated at the level of mRNA stability, translation and protein secretion [135,136,137,138,139,140,141].

## 6. Gelatinase B/MMP-9 Expression, Bioavailability, Activity and Endogenous Inhibitors

Gelatinase B/MMP-9 expression is up-regulated *in vitro* by pro-inflammatory cytokines and PKC activators in human melanoma, neuroblastoma, teratocarcinoma, lung cancer and fibrosarcoma cells [15,16] and in rabbit fibroblasts [131], by chemokines in prostate cancer cells [142] and by growth factors, such as TGFβ in human breast cancer cells [143], EGF in human prostate [144], squamous cell carcinoma [145] and renal carcinoma cells [146], HGF in colon [147], renal [148], hepatocellular carcinoma [149], mesothelioma [150], lung cancer [151] and pancreatic tumour cells [152], by FGF in rabbit fibroblasts [131], human osteosarcoma cells [153], human bladder cancer cells [154] and human breast cancer cells [155,156], by neuropeptides in prostate cancer cell lines [157] and by haemoglobin in malignant melanoma and bladder cancer cells [158]. Gelatinase B/MMP-9 is also induced in neuroblastoma cells in association with spontaneous epithelial to neuroblast phenotype conversion and following treatment with all-*trans*-retinoic acid [123,124] and released from IL-8 stimulated neutrophils [159].

Gelatinase B/MMP-9 enzymatic activity is inhibited by the universal systemic protease inhibitor α2-macrogloblin [160], members of the tissue inhibitors of metalloproteinases (TIMPs) family [161,162] and is also antagonized by its own isolated hemopexin domain [41,163]. TIMPs 1–4 are 20–30 kDa glycoprotein MMP inhibitors that depend upon disulphide bridges between 6 cysteine pairs for their inhibitory activity [161,162,164]. TIMP-1 exhibits a unique binding interaction with gelatinase B/MMP-9 and, with the exception of human neutrophils, exhibits a high level of coordinated expression with TIMP-1, is frequently secreted as a TIMP-1/gelatinase B/MMP-9 complex and binds gelatinase B/MMP-9 with high affinity, in contrast to TIMP-2 and TIMP-3 [12,15,41,123,157,162]. The interaction between pro-form gelatinase B/MMP-9 and TIMP-1 involves the *C*-terminal domains of both proteins and in this form TIMP-1 is available to inhibit other MMPs. Upon gelatinase B/MMP-9 activation, TIMP-1 inhibits gelatinase B/MMP-9 catalytic activity through *N*-terminal interaction with the gelatinase B/MMP-9 catalytic site, with inhibition facilitated by the gelatinase B/MMP-9 *C*-terminus, since it does not readily occur in gelatinase B/MMP-9 C-terminus deletion mutants. In contrast to TIMP-1, TIMP-2 inhibition of gelatinase B/MMP-9 depends upon the *N*-terminal domain, but does not involve *C*-terminal interaction and is less effective that TIMP-1. TIMP-3 is a matrix-associated inhibitor that interacts with and inhibits gelatinase B/MMP-9 to a lesser extent than either TIMP-1 or TIMP-2 [161,162].

The bioavailability of gelatinase B/MMP-9 is regulated by forming complexes with low-density lipoprotein receptor-related proteins (LRP)-1 and LRP2 via functional endocytosis, promoting intracellular gelatinase B/MMP-9 uptake and leupeptin-sensitive degradation [49,165]. Autocatalytic gelatinase B/MMP-9 degradation is prevented when monomeric gelatinase B/MMP-9 is complexed with Neutrophil gelatinase-associated lipocalin (NGAL) in an interaction that does not result in gelatinase B/MMP-9 inhibition, effectively prolonging enzymatic activity [166,167].

### The Gelatinase B/MMP-9/TIMP-1 Protease-Antiprotease Equilibrium

Mechanisms that alter the equilibrium between gelatinase B/MMP-9 and its TIMP-1 inhibitor in favour of protease activity, facilitate gelatinase B/MMP-9 involvement in tumour pathology, and include differential expression, evasion from TIMP inhibition, and TIMP-1 inactivation.

Gelatinase B/MMP-9 and TIMP-1 are frequently co-ordinately expressed in a large number of cell types and secreted as a pro-gelatinase B/MMP-9/TIMP-1 complex [12,15]. The tumor environment is however complex, and composed of tumor, stromal and inflammatory elements that also contribute to the modulation of this important equilibrium. Tumor infiltrating neutrophils release gelatinase B/MMP-9 in TIMP-free form, facilitating tumor-associated differential gelatinase B/MMP-9 and TIMP-1 expression [168,169] Furthermore, the differential up-regulation of gelatinase B/MMP-9 but not TIMP-1 expression has been reported in human ovarian cancer [170], skin cancer [171], squamous cell carcinoma of the hypopharynx [172] and colon and rectal tumours *in vivo* [173], and has also been demonstrated in malignant melanomas induced in metallothionin/RET transgenic mice [174]. *In vitro*, differential up-regulation of gelatinase B/MMP-9 but not TIMP-1 expression characterises PC-3 prostate tumor cell/stromal cell co-cultures and endothelial cells co-cultured with fibroblasts [175,176], cervical carcinoma cells in response to CD40L activation [177], human head and neck squamous carcinoma cells in response to c-erbB ligands [178], spontaneous epithelial to neuroblast transformation of human neuroblastoma cells [123], retinoic acid treatment of differentiation resistant human neuroblastoma cells [124], peroxiredoxin expression in metastatic human MDA-MB-231 breast cancer cells [179], thioredoxin expression in human MDA-MB-231 breast cancer cells [132] and bFGF treatment of human retinoblastoma cells [180].

Differential gelatinase B/MMP-9 and TIMP-1 regulation may also involve promoter SNPs and/or 3’-UTR micro RNA binding sites. Indeed, gelatinase B/MMP-9 SNPs that augment gelatinase B/MMP-9 expression have been associated with increased risk of different forms of cancer (See Section Gelatinase B/MMP-9 SNPs on page 241), as have altered levels of miRs that bind miR binding sites within the 3'-UTR region of gelatinase B/MMP-9. The miRs -211, 491-5p and 885-5p target and inhibit gelatinase B/MMP-9 expression and are down regulated in human glioblastoma multiforme, in association with increased gelatinase B/MMP-9 expression [17,18], and miR-19a has been reported to regulate gelatinase B/MMP-9 expression in colon cancer cells [181]. Furthermore, a recent report has shown that miR-17 targets the TIMP-1 protein-coding region and its inhibition enhances TIMP-1 expression and decreases gelatinase B/MMP-9 activity [182]. It is likely, therefore, that altered patterns of miR expression may also facilitate the differential expression of gelatinase B/MMP-9 and TIMP-1 in malignant tumours.

Extracellular activation of the thioredoxin redox system, up-regulated in malignant tumours, has been shown to inhibit TIMP but not MMP activity *in vitro* and in models of human neuroblastoma and UV irradiated dermal fibroblasts [164,183]. Furthermore, the myeloperoxidase/H_2_0_2_/hypochlorous acid (HOCl) system of inflammation induces the oxidative inactivation of TIMPs, whilst promoting the activation of MMPs, at concentrations found during inflammation [184,185], providing mechanisms through which the gelatinase B/MMP-9/TIMP equilibrium within tumours can be altered in favour of proteolytic activity even under conditions of high level TIMP expression [186]. TIMP MMP-inhibitory activity, furthermore, can be destroyed by neutrophil elastase, trypsin and α-chymotrypsin, all of which activate gelatinase B/MMP-9 [12,187,188], providing an additional mechanism for irreversible TIMP inhibition combined with gelatinase B/MMP-9 activation within inflammatory tumour environments and also environments such as the pancreas, in which trypsin and trypsin-like enzymes are expressed [189]. Finally, truncated gelatinase B/MMP-9 isoforms generated by enzymatic digestion or present on the cell surface of human leukemic cells have been shown to escape TIMP inhibition (see Section 3.4).

## 7. Gelatinase B/MMP-9, Tumour Initiation/Promotion and Genetic Instability

**Figure 2:**
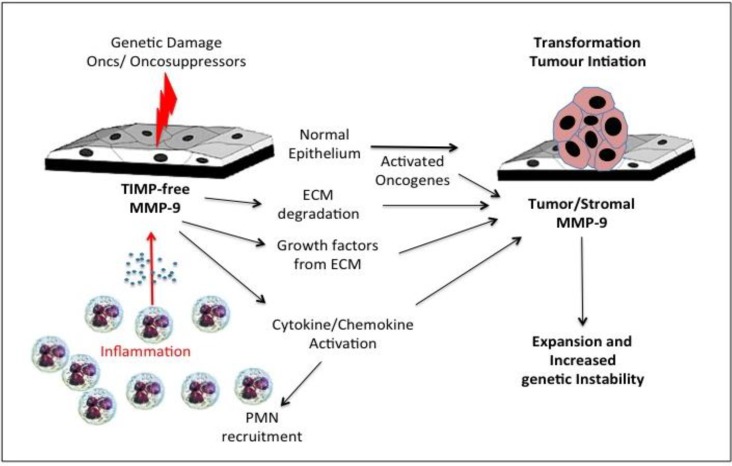
Representation of the role of inflammatory polymorphonuclear leukocyte (PMN)-derived tissue inhibitor of metalloproteinase (TIMP)-free gelatinase B/MMP-9 in tumour initiation and promotion of genetic instability through degradation of extracellular matrices (ECM) release and activation of cytokines, chemokines and growth factors.

Potential pro-oncogenic roles for gelatinase B/MMP-9 have been reported, implicating gelatinase B/MMP-9 in neoplastic transformation, tumour initiation/promotion and genetic instability (Figure 2). Gelatinase B/MMP-9 localises to the nucleus, despite lack of classical nuclear localisation signal [190,191] and nuclear gelatinase activity associates with increased levels of DNA fragmentation [192,193,194]. Indeed, nuclear gelatinase degrades the nuclear matrix protein poly-ADP-ribose-polymerase (PARP), hindering DNA repair [193,195]. Furthermore, gelatinase B/MMP-9 binds the DNA damage heterodimer Ku70/80, providing a potential mechanism for its nuclear translocation [196]. Nuclear gelatinase B/MMP-9 has been reported in human gliomas, astrocytomas and neuroblastomas [197,198].

Gelatinase B/MMP-9 has been reported to protect colorectal cancer cells against microsatellite instability, with reduced gelatinase B/MMP-9 activity associated with increased microsatellite instability. This has been attributed to inhibitory mutations within the promoter of the gelatinase B/MMP-9 activator MMP-3 and is associated with better prognosis [199,200,201]. Microsatellite instability, furthermore, down-regulates gelatinase B/MMP-9 expression by introducing polymorphisms that reduce the number of (CA)n repeats within gelatinase B/MMP-9 regulatory domain to below 22 [28].

A role for inflammatory neutrophil-derived gelatinase B/MMP-9 in intestinal adenoma initiation has been described in heterozygous APC (APC-min) knockout mice, with a 40% reduction in adenoma formation observed upon gelatinase B/MMP-9 knockout [202]. Increased gelatinase B/MMP-9 activity provided by inflammatory neutrophils, furthermore, augments neutrophil recruitment via gelatinase B/MMP-9-mediated degradation and super-activation of IL-8 [106], augmenting neutrophil-mediated genetic instability [106,203]. Gelatinase B/MMP-9 also induces Rac1b alternative splice variant expression, which promotes chromosomal instability by increased reactive oxygen species levels and activating Snail-mediated transcription, resulting in increased oxidative DNA damage [204,205].

Gelatinase B/MMP-9 has also been reported to promote liver tumour initiation by the proteolytic release and activation of matrix-associated TGFβ and VEGF [206], and in human mammary epithelial cells induces cell surface expression of the HER2/Neu oncoprotein, inhibiting apoptosis and shifting normal mammary cells towards a transformed phenotype, in the presence of oestrogen [207].

In contrast, gelatinase B/MMP-9 optimises non-homologous end joining (NHEJ) DNA repair in human glioma cells. Indeed, down-regulation of gelatinase B/MMP-9 expression, combined with either urokinase or cathepsin B, delays DNA repair by lowering KU70/80 recruitment to damaged DNA. This reduces NHEJ DNA repair function, increases the levels of DNA damage and promotes apoptosis [208].

## 8. Gelatinase B/MMP-9 and Tumour Initiating Cell Proliferation and Expansion

Clonal expansion of transformed cells is also an essential step in tumour progression and is facilitated by inflammation and involves a change in equilibrium between proliferation, apoptosis and angiogenesis [209,210].

In the heterozygous APC knockout mouse model (APC-min), neutrophil-derived gelatinase B/MMP-9 stimulates adenoma initiating cell proliferation, promoting adenoma expansion, and implicating gelatinase B/MMP-9 in the expansion of tumour cell populations that lack full APC function. It is likely that this involves gelatinase B/MMP-9-mediated release and activation of non-matrix cytokines, such as TNFα and IL-1β and matrix-associated growth factors, such as VEGF, TGFβ and FGFs and/or the degradation of growth inhibitors [211,212,213,214]. Indeed, gelatinase B/MMP-9 degrades IGF-BPs augmenting the circulating levels of IGF, promoting astrocytoma growth [215], and increasing circulating VEGF and EGF levels [216], which also promote adenoma cell proliferation in APC-min mice. Furthermore, transcriptional silencing of gelatinase B/MMP-9 inhibits human glioma cells proliferation [208] and Wnt signalling induced by hypoxia stimulating gelatinase B/MMP-9 expression and promotes neural stem cell proliferation [217], suggesting that a hypoxia/Wnt/gelatinase B/MMP-9 axis may also promote proliferation of the cancer stem/progenitor cell component of neural-related tumours.

## 9. Gelatinase B/MMP-9, Stem Cells and the Cancer Stem Cell Niche

The stem cell niche is a unique, specialised location responsible for maintaining stem cells. Stem cells within the niche are anchored by intracellular and cell matrix adhesive interactions, which regulate stem cell numbers, stem cell self-renewal and potentially asymmetrical stem cell division. Normal stem cells and cancer stem cells exhibit similar behaviour [218,219]. Cancer stem cell niches have been identified in tumours and implicated in tumour heterogeneity, metastatic progression and therapeutic resistance, and are regulated by conditions within the tumour and promoted by tumour associated stress such as hypoxia [220,221]. Gelatinase B/MMP-9 has been implicated in regulating stem cell niche behaviour and within the bone marrow, degrading extracellular matrices within the stem cell niche, resulting in the activation and mobilisation of haemopoetic stem cells. This is facilitated by the conversion of stem cell factor from its membrane bound to free form, promoting c-Kit receptor-mediated stem cell proliferation [90]. Gelatinase B/MMP-9 also releases circulating endothelial precursor stem cells from the bone marrow, contributing to angiogenesis [90]. Interaction between stroma-derived factor (SDF)-1 and the chemokine receptor CXCR4 is essential for stem/progenitor cell function [222] and induces gelatinase B/MMP-9 expression. A similar interaction induces gelatinase B/MMP-9 expression in cancer cells, promoting dissemination and metastasis to bone [222,223,224]. Wnt signalling induces gelatinase B/MMP-9 expression and maintains stem cell niche integrity [225,226]. Wnt signalling is up regulated in cancer, and also stimulates cancer stem cell proliferation, resistance to apoptosis, tumour invasion and metastasis [227,228,229]. Furthermore, Wnt-induced gelatinase B/MMP-9 expression has been implicated in embryonic neural stem cells proliferation in conditions of hypoxia [217], a similar mechanism may, therefore, regulate cancer stem cells proliferation within neural tumours [218].

## 10. Gelatinase B/MMP-9 and Epithelial to Mesenchymal Transition (EMT)

Epithelial to mesenchymal transition (EMT) represents the conversion of polarized immotile epithelial cells into motile mesenchymal progenitor cells. This mechanism is important in development (type 1), normal wound healing or pathological fibrosis (type 2) and in the metastatic transformation of cancer cells (type 3) [230]. Type 3 EMT is fundamental for tumour progression to metastasis, and is either re-activated in de-differentiated epithelial cancer cells or activated in epithelial cancer stem cells, inducing a more motile and invasive phenotype [228]. It may also be transient, with metastatic cells reverting back to an epithelial phenotype at destination. 

**Figure 3:**
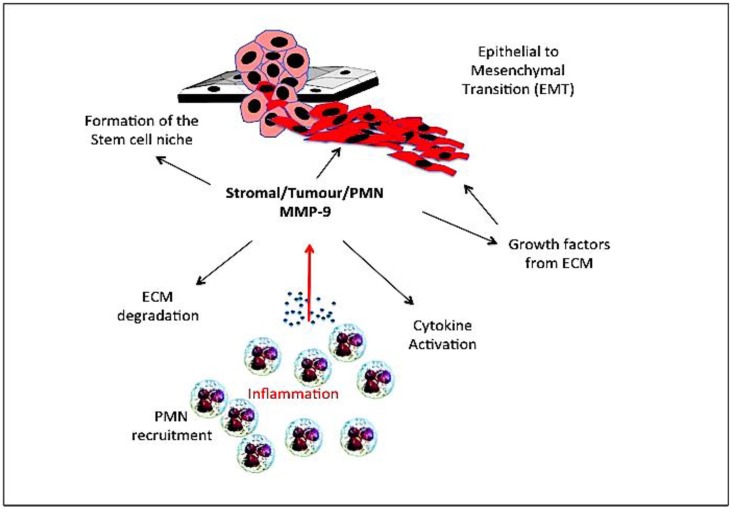
Representation of the role played by inflammatory polymorphonuclear leukocyte (PMN), stromal and tumour cell-derived gelatinase B/MMP-9 in epithelial-mesenchymal transition (EMT) and formation of the stem cell niche through degradation of extracellular matrices (ECM) release and activation of cytokines and growth factors.

Gelatinase B/MMP-9 is an important EMT-related gene, and is not only a consequence but also a cause of EMT (Figure 3). Gelatinase B/MMP-9 cooperates with Snail transcription factor to induce EMT in epidermoid carcinoma cells [231], is involved in medulloblastoma cell EMT [232], is induced by Twist transcription factor an essential inducer of EMT [233,234,235] and by krupple like factor (KLF)-8, a critical component of FAK-regulated breast cancer EMT, which induces gelatinase B/MMP-9 expression in human breast cancer cells, promoting migration, invasion, angiogenesis and metastasis [234,235,236]. EMT in gastric cancer involves a Shh/PI3K/Akt/gelatinase B/MMP-9 pathway, which promotes metastatic dissemination to lymph nodes [237]. In human neuroblastoma cells, spontaneous EMT-like phenotypic conversion from a less invasive epithelial to more invasive neuroblast phenotype, associates with the induction of gelatinase B/MMP-9 expression and increased gelatinase B/MMP-9-mediated invasion [123].

## 11. Gelatinase B/MMP-9 and Cancer-Related Inflammation

Inflammation is now considered to be a hallmark of tumour progression, and regulates tumour-associated angiogenesis, tumour cell proliferation, invasion and metastasis [209,210]. Gelatinase B/MMP-9 is considered to be a tuner and amplifier of inflammatory and immune functions [106,238] and is up regulated by pro-inflammatory cytokines such as TNFα, IL-1β, IL-6 and TGFβ in a wide variety of human tumour cells, stromal and endothelial cells [12,13,15]. Gelatinase B/MMP-9 activates pro-inflammatory cytokines TNFα and IL-1β, increases the activity of chemokines CXCL1, CXCL4, CXCL7 and CXCL8, releases TGFβ from matrix stores, is released by activated neutrophils in TIMP-1-free form and acts as a nanomolar effector of tumour associated inflammation [12,13]. Neutrophil-derived gelatinase B/MMP-9 also interacts with neutrophil NGAL, which prevent autolytic gelatinase B/MMP-9 processing but does not impair gelatinase B/MMP-9 activity, promoting tumour progression [130,161]. CXCL8 interaction with the chemokine receptor CXCR2 induces gelatinase B/MMP-9 release from neutrophils [239], and activation of the chemokine receptor CXCR4 up-regulates gelatinase B/MMP-9 expression in prostate tumour cells, promoting invasion and metastasis [240]. Furthermore, myeloperoxidase/H_2_O_2_/HOCl system activation in neutrophils activates gelatinase B/MMP-9 and inhibits TIMP activity [184,185]. Gelatinase B/MMP-9, therefore, exhibits an integral relationship with tumour-associated inflammation. Indeed, the inhibition of gelatinase B/MMP-9 expression by inhibitors of pro-inflammatory cyclooxygenase-2 reduces tumour cell proliferation, invasion and metastasis [241,242]. In addition to its relationship with neutrophils, gelatinase B/MMP-9 also promotes macrophage and tumour cell invasion by cleaving the TGF-β-induced protein βig-h3, releasing it from the extracellular matrix, whereas non-degraded βig-h3 inhibits cell migration by promoting cell-cell contact and cell-extracellular matrix interactions [243].

In contrast to its tumour-promoting effects, high-level gelatinase B/MMP-9 expression has also been reported to promote tumour regression in a breast cancer model, augmenting neutrophil infiltration and promoting tumour-associated macrophage anti-tumour activity [244]. 

## 12. Gelatinase B/MMP-9 and Angiogenesis

The formation of new blood vessels is a highly orchestrated process that depends upon mitogenic and non-mitogenic angiogenic factors and involves matrix remodelling, cell migration, and regulated adhesive interactions between vascular cells and with the matrix. Tumour neovascularisation is fundamental for primary tumour expansion, metastatic progression and metastatic growth, and occurs via processes including sprouting angiogenesis, vasculogenesis, co-option inter-susception and/or vascular mimicry. Unlike normal vessels, blood vessels within tumours are abnormal, immature and inflammatory in nature [245].

**Figure 4:**
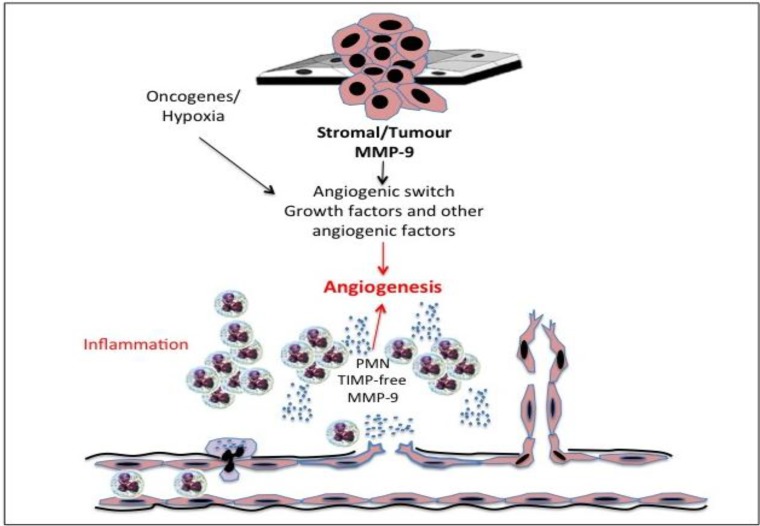
Representation of the roles played by inflammatory polymorphonuclear leukocyte (PMN)-derived tissue inhibitor of metalloproteinase (TIMP)-free gelatinase B/MMP-9, gelatinase B/MMP-9 of stromal and tumour origin, oncogenes and hypoxia in activating the tumour angiogenic switch required for tumour progression.

Gelatinase B/MMP-9 is a critical pro-angiogenic molecule [246] and triggers the “angiogenic switch” in the quiescent vasculature [247,248] (Figure 4). Both host inflammatory and vascular gelatinase B/MMP-9 has been shown to be crucial for the development of the tumour angiogenic vasculature in models of pancreatic, ovarian and skin cancer [170,211,249]. Neutrophil gelatinase B/MMP-9 regulates pericyte proliferation, apoptosis and recruitment during angiogenesis [170] and mobilises the recruitment of bone marrow-derived angiogenic precursors to the tumour stroma enhancing the tumour angiogenic and vasculargenic process [90,250,251,252]. Gelatinase B/MMP-9 also triggers “the angiogenic switch” by mobilising and activating angiogenic mitogens from matrix stores at the onset of tumour-associated angiogenesis [169,211,251,253]. This process is facilitated by the release of TIMP-1-free gelatinase B/MMP-9 from neutrophils, which acts as an exceptionally potent nanomolar angiogenic factor, releasing both FGF and VEGF from matrices [169,254]. 

The gelatinase B/MMP-9/VEGF axis not only supports angiogenesis but also promotes hyperactive haematopoiesis, [255,256], which also promotes tumour progression by expanding myeloid-derived suppressors that suppress T-lymphocyte proliferation and activation, promoting tumour evasion of immune surveillance [257,258,259]. Mouse gelatinase B/MMP-9 has been shown to cleave VEGF to a truncated VEGF_121_ form that promotes irregular neovascularisation by altering interactions with heparan sulphate and other matrix components [118]. This, however, does not appear to extend to human gelatinase B/MMP-9 [80].

In addition to promoting pericyte recruitment, gelatinase B/MMP-9 also promotes the recruitment of pro-angiogenic monocytes and CD34^+^ endothelial cell progenitors, which express VE-cadherin and VEGFR2, to tumours, markedly influencing angiogenesis [252,260,261,262,263] and induces the release of circulating endothelial precursor stem cells from the bone marrow by degrading c-kit ligand, contributing to both angiogenesis and vasculogenesis [90]. In human neuroblastoma, gelatinase B/MMP-9 recruits bone marrow-derived leukocytes and support cells to tumour vessels, regulating vessel maturation [264] and the VEGF/gelatinase B/MMP-9 axis has been implicated in the robust angiogenic response associated with TrkAIII oncogene promotion of neuroblastoma tumorigenicity [265]. In gelatinase B/MMP-9 knockout mice, impaired vascularisation associates with reduced pericyte-recruitment [266] and vascular pericytes have been shown to express gelatinase B/MMP-9 in human breast tumours [267]. In general, knockout technology has implicated MMP9 in skeletal growth plate vascularisation [268] and in bone marrow derived CD11b^+^ myelomonocytic cell-mediated vasculogenesis in irradiated tumour tissues, with the absence of gelatinase B/MMP-9 associated with small tumours containing mature vessels [269]. Gelatinase B/MMP-9 has also been implicated in capillary branching during ischemia-induced revascularisation [270]. 

Although, bone marrow-cell derived gelatinase B/MMP-9 appears to be sufficient for tumour vasculogenesis, it is not essential and can be substituted by gelatinase B/MMP-9 from either stromal, smooth muscle or tumour cell components. Indeed, fibroblast gelatinase B/MMP-9 enhances endothelial cell survival and function [271], gelatinase B/MMP-9 from circulating macrophages promotes angiogenesis in a model of pancreatic cancer [272] and increased tumour cell gelatinase B/MMP-9 promotes angiogenesis in a model of neuroblastoma [265].

Tumour-associated hypoxia is a major stimulus for angiogenesis and hypoxia exhibits an overall tendency to increase vascular gelatinase B/MMP-9 expression [273,274]. Neovascularization induced by hypoxia involves Nox2-derived ROS-mediated gelatinase B/MMP-9 activation [275] and under conditions of hypoxia gelatinase B/MMP-9 modulates endothelial cell behaviour, promoting human microvascular endothelial cell invasive and angiogenic capacity [276]. Inflammatory cytokines TNFα, IL-17 and IL-18 promote gelatinase B/MMP-9 regulated migration of pericyte and vascular smooth muscle cell migration during angiogenesis [277,278,279,280] and gelatinase B/MMP-9 knockout impairs both pericyte and vascular smooth muscle cell migration, decreasing intimal vascular hyperplasia [281,282]. Furthermore, in addition to mobilising bone marrow CD34^+^ stem cells, gelatinase B/MMP-9 also promotes endothelial cell progenitor proliferation [262,263], degrades basement membrane type IV collagen, exposing cryptic αVβ3 integrin binding sites that promote angiogenesis [283] and releases VEGF from matrices in angiogenic islets, promoting angiogenesis [211,251].

In contrast to autocrine angiogenesis stimulating effects of gelatinase B/MMP-9 [212], gelatinase B/MMP-9 also exhibits capacity to negatively regulate angiogenesis by producing endogenous anti-angiogenic factors such as endostatin, tumstatin and angiostatin [72,112,284,285]. Endostatin, formed by gelatinase B/MMP-9 digestion of the type XVIII collagen α1 chain [72], blocks VEGFR2 and α5β1-mediated angiogenesis, inhibits gelatinase B/MMP-9 activity [286,287,288,289] and reduces metastasis in patients with high-grade transitional cell carcinoma of the bladder [290]. Tumstatin, formed by gelatinase B/MMP-9 digestion of the collagen IV α3 chain, inhibits endothelial cells proliferation and promotes αVβ3-mediated endothelial cell apoptosis [235,239]. Angiostatin, formed by gelatinase B/MMP-9 digestion of plasminogen and plasmin [112,113], acts as a competitive inhibitor of tissue-type plasminogen activator and single chain urokinase-mediated plasminogen activation, inhibits plasmin-mediated laminin degradation, impairs plasminogen association with the tumour cell surface, and inhibits plasmin-dependent tumour invasion and angiogenesis [113,285]. The gelatinase B/MMP-9 hemopexin domain, which can be generated by plasmin-mediated degradation of cryptic sites within the gelatinase B/MMP-9 catalytic domain, also inhibits gelatinase B/MMP-9 activity and angiogenesis [41,163,291].

Other interactions involving gelatinase B/MMP-9 that regulate angiogenesis include: thrombospondin-1 induction of gelatinase B/MMP-9 expression but inhibition of gelatinase B/MMP-9 activation [292], and gelatinase B/MMP-9 interaction with syndecan-1, which promotes syndecan-1 shedding and enhances medulloblastoma cells tube forming capacity. This involves an gelatinase B/MMP-9/syndecan-1/miR-494 regulatory loop, involved in regulating irradiation-induced angiogenesis, in which syndecan and gelatinase B/MMP-9 activity negatively feedback to regulate miR494 expression, which promotes angiogenesis [293]. Interaction between angiogenic endothelial cells and prostate cancer cells has also been reported to activate an IL-6/androgen receptor/TGFβ/gelatinase B/MMP-9 signal pathway that augments prostate cancer invasion in association with angiogenesis [294]. 

Angiogenic factors stimulate and/or associate with gelatinase B/MMP-9 expression. Ang2 expression correlates with that of gelatinase B/MMP-9 [295]. VEGF induces gelatinase B/MMP-9 expression in vascular cells and some malignant tumour cell types [296,297,298]. Hypoxia induces VEGF-A expression resulting in the recruitment of pro-angiogenic neutrophils that deliver the gelatinase B/MMP-9 and trigger the “angiogenic switch” [299]. Angiogenic fibroblast growth factors induce gelatinase B/MMP-9 expression in tumour and stromal tissues [153,300,301], and agents that inhibit MMP9 expression and/or gelatinase B/MMP-9 activity, such as DMBT [302], propofol [303], secreted protein acidic and rich in cysteine (SPARC) [304], S100A4 [305], xylitol [306], wortmanin [235], BMP4 [307], and aloe emodin [308], down regulate angiogenesis in different models.

Vasculargenic mimicry by tumour cells has been equated to tumour-associated angiogenesis [309]. Gelatinase B/MMP-9 has been also implicated in the vasculargenic mimicry exhibited by Adriamycin-resistant MCF-7 breast cancer cells, promoting tubular network formation through a VEGF receptors VEGFR-2 and VEGFR-3-mediated mechanism, implicating gelatinase B/MMP-9 in tumour-associated vascular mimicry [310].

### Gelatinase B/MMP-9 and Lymphangiogenesis

Lymphagiogenesis is also an important component of tumour progression, with lymphatic vessels providing important routes for metastatic dissemination [311,312]. Although gelatinase B/MMP-9 is not required for normal skin lymphangiogenesis [313], tumour induced lymphangiogenesis has been reported to involve a sonic hedgehog/PI3K/Akt/gelatinase B/MMP-9 pathway, leading to lymph node metastases in gastric cancer [237]. Furthermore, neutrophil-derived gelatinase B/MMP-9 has been implicated in inflammation-associated lymphangiogenesis, promoting VEGF-A bioavailability and bioactivity [314] and, together with VEGF-C, has been implicated in lymphangiogenesis and lymph node metastasis in breast cancer [315].

## 13. Gelatinase B/MMP-9 and Disruption of Tissue Architecture

The loss of tissue architecture is one of the earliest hallmarks of premalignant epithelial cancer and results in tumour proliferation, local invasion and angiogenesis [316]. In malignant breast cancer, tumour cells loose their capacity to form ordered structures and proliferate as disorganised colonies [317]. Raf/MEK/ERK-mediated induction of gelatinase B/MMP-9 expression results in the destruction of breast tissue architecture, during breast cancer initiation, by degrading basement membrane laminin and destroying basement membrane integrity. This results in de-regulated tissue polarity and the loss of growth control (Figure 4). Gelatinase B/MMP-9 inhibition halts this process by preserving basement membrane integrity, which in turn reverses phenotype, arrests growth and re-establishes a differentiated acinar polarity [60].

## 14. Gelatinase B/MMP-9 Induction of Intracellular Signalling

Gelatinase B/MMP-9 interacts with the cellular surface through either Ku70/80 [196], CD44 [48,318] or via integrins [318]. Gelatinase B/MMP-9 interacts with αL, β5, α4 and β1 subunits through its catalytic site and interacts with CD44, α4, β5 and β1 subunits through the hemopexin domain [319,320]. These interactions stimulate migration, promote survival, increase both proteolytic and non-proteolytic invasion [318,320,321,322] and promote angiogenesis [169]. Signalling events in these interactions include, JNK involvement in gelatinase B/MMP-9-mediated dendritic cell migration, which is blocked by the JNK inhibitor SP600125 [323], and MAPK and IP3K involvement in gelatinase B/MMP-9-induced endothelial cell migration, which is blocked by the MAPK inhibitor PD98059 and by the IP3K inhibitor LY294002. Apoptosis in medulloblastoma, associated with loss of gelatinase B/MMP-9 expression, involves β1 integrin, ERK signalling and NF-κB activation [324,325]. Gelatinase B/MMP-9 interaction with α4β1 and CD44 induces survival signalling in CLL cells, activating lyn kinase, phosphorylating STAT and up-regulating Mcl-1 expression [322]. Interaction between gelatinase B/MMP-9 and CD44 results in EGF receptor activation and signalling through ERK, Akt and FAK, which promotes tumour cell invasion and migration [326], with FAK coordinating adhesion, polarisation, migration, invasion, survival and death [327].

## 15. Gelatinase B/MMP-9, Tumour Cell Invasion and Motility

**Figure 5:**
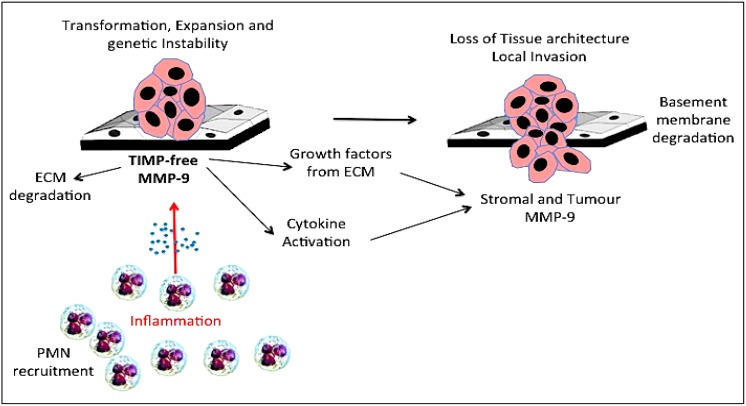
Representation of the roles played by inflammatory polymorphonuclear leukocyte (PMN)-derived tissue inhibitor of metalloproteinase (TIMP)-free gelatinase B/MMP-9, gelatinase B/MMP-9 of stromal and tumour origin, in the loss of tissue architecture and local invasion associated with tumour progression.

Within the tumour context, gelatinase B/MMP-9 was originally identified as a novel type IV collagenolytic protease secreted by metastatic Ras transformed fibroblasts and implicated in basement membrane disruption required for tumour invasion and metastasis [6,7]. Although it remains debatable as to whether gelatinase B/MMP-9 alone can effectively degrade type IV collagen within the context of an insoluble basement membrane matrix, gelatinase B/MMP-9 promotes invasion by disrupting basement membrane structure by degrading basement membrane laminin and type IV collagen, in concert with other enzyme systems [8,57,59,60]. Interaction between tumour cells and stroma at the invasive edge regulates gelatinase B/MMP-9 expression, which combined with gelatinase B/MMP-9 released by tumour-associated neutrophils and macrophages, increases invasiveness [175,328,329,330] (Figure 5).

Tumor cell invasion is, however, a complex process that depends upon alterations in protein expression, interaction between tumour, inflammatory and stromal cells, altered intercellular and extracellular adhesive interactions, and changes in the tumour microenvironment. It is regulated by pro-inflammatory cytokines, chemokines, growth factors, matrix components, integrin and non-integrin receptors, proteases and inhibitors, and depends upon the cellular motile response. Cellular motility is achieved by different mechanisms and can reversibly switch between mesenchymal and amoeboid migration, which promote invasion as either single cells or collective chains, sheets, columns, tubes or clusters [331].

Protease involvement in migration and invasion is relatively restricted to mesenchymal motility, whereas amoeboid motility does not require proteolytic activity but involves a high level of cellular deformability, low affinity substrate binding and cycles of morphological contraction and expansion [331,332]. Mesenchymal migration, on the other hand, requires high affinity binding to integrin and non-integrin receptors. During mesenchymal migration, integrin or non-integrin receptors concentrate to membrane lamellipodia, filopodia, pseudopodia and invadopodia, promoting adapter protein-mediated intracellular interaction with the actin cytoskeleton. This results in the formation of focal contacts and adhesions with extracellular matrix components, the maturation of which activates intracellular focal adhesion kinases (FAKs) that form transient signalling complexes with Src kinases, promoting movement by inducing the turnover of focal contact providing the propulsive force for movement by continually modifying of cell-matrix interactions. These events depend upon proteolytic activity and involve the fibronectin integrin receptors α5β1 or αVβ6, the laminin integrin receptors α6β1 or α6β4, the fibronectin/vitronectin integrin receptor αVβ3 and the fibrillar collagen receptor α2β1 [331].

Gelatinase B/MMP-9 regulates mesenchymal migration, co-localises with integrins at lamellipodia on migrating cells [333] and co-operates with αVβ3 integrin to increase breast cancer cell migration and metastatic capacity [334]. FAK-Src signalling through JNK transcriptionally upregulates gelatinase B/MMP-9 expression, promoting gelatinase B/MMP-9-mediated invasion [335,336,337], and αVβ6 or α5β1 interaction with fibronectin also increases gelatinase B/MMP-9 expression, and gelatinase B/MMP-9-mediated migration and invasion of squamous cell carcinoma and melanoma cells [338,339,340]. Fibronectin also induces gelatinase B/MMP-9 expression in ovarian cancer cells through FAK and ras activation [335] and laminin has been shown to up-regulate gelatinase B/MMP-9 expression in macrophages and in A2058 melanoma cells but not in other malignant tumour cells [341,342]. Gelatinase B/MMP-9 promotes chain migration of neural crest cells [343] and collective migration of epithelial cancer cells, in association with EMT [344]. 

Gelatinase B/MMP-9 also interacts with the non-integrin receptor CD44, concentrating gelatinase B/MMP-9 to cell extensions, which control the turnover of adhesive interactions and extracellular matrix degradation required for motility, in a coordinated process that also involves ezrin, actin and Krp1 [336]. Gelatinase B/MMP-9 interaction with CD44 also promotes breast cancer cell migration and invasion in association with EGFR activation [48]. An *N*-cadherin/FGFR/gelatinase B/MMP-9 axis has been implicated in breast cancer cell invasion and metastasis, bypassing E-cadherin invasion and metastasis suppressing signals [155]. Gelatinase B/MMP-9 degradation of protease nexin-1 has also been implicated in a novel pathway through which gelatinase B/MMP-9 regulates tumour cell invasion, impairing the capacity of nexin to bind and down-regulate the activity of uPA [345]. 

In contrast to these reports, gelatinase B/MMP-9 has also been shown to degrade the β4 component of α6β4 integrin, de-regulating sheet migration of epithelial cells [346]. Furthermore, gelatinase B/MMP-9 interaction with α4β1 integrin and CD44 on the surface of chronic leukemic cells has been shown to inhibit migration in response to chemotactic gradients [318]. Tumour cells, furthermore, can undergo mesenchymal to amoeboid transition (MAT). Gelatinase B/MMP-9 is not required for amoeboid movement through 3D interstitial matrices [332] and MAT has been shown to increase metastatic capacity in association with reduced gelatinase B/MMP-9 expression [347]. 

### Gelatinase B/MMP-9 and Primary Tumour Cell Escape

In order to escape the constraints of the primary tumour, tumor cells invade, move and alter their adhesive interactions. Chemotactic motile responses may direct tumour cells to lymphatic or blood vessels and tumour interaction, with tumour associated macrophages facilitating directional movement within tumours [348]. HGF activation of tumour cell c-met induces gelatinase B/MMP-9 expression, increasing tumor cell motility and scattering [349].

## 16. Gelatinase B/MMP-9 and Immunological Surveillance

The capacity to evade elimination by immunological/inflammatory mechanisms is an essential feature of tumour progression to metastasis. Gelatinase B/MMP-9 is an important regulator of both innate and tumour immune responses [12]. This is illustrated in gelatinase B/MMP-9 knockout mice, which do not resolve contact hypersensitivity reactions, implicating gelatinase B/MMP-9 in the down regulation of the immune response [350], suggesting an analogous role for gelatinase B/MMP-9 in cancer. In support of this, gelatinase B/MMP-9 expression associates with that of anti-pathogen immune-response-related genes in late stage compared to early stage lung tumours, although it remains to be determined whether any of these represent novel gelatinase B/MMP-9 substrates [351]. Gelatinase B/MMP-9 degrades ICAM-1, down-regulating leukocyte homing [80] and promotes evasion of the immune system by chronic myeloid leukemia cells by solubilizing cell membrane ICAM-1 [352]. Gelatinase B/MMP-9 degrades the IL-2 receptor α, repressing activation and proliferation of tumour infiltrating T-lymphocytes in cervical cancer [85,86]. Gelatinase B/MMP-9 degrades Surfactant protein D (SP-D), an important component of innate immune defence, leading to loss of innate immune function, limiting SP-D involvement in tumour immunology and renders oncology patients more susceptible to infection [353]. Gelatinase B/MMP-9 digests C1q complement component at a site required for interaction with the C1qR_02_ receptor, repressing C1q/C1qR_02_ involvement in tumour immunology [109] and may also degrade complement component C1r [65]. C5a induces the expression of the gelatinase B/MMP-9 stimulator IL-1β in monocytes [15,354] and the complement membrane attack complex induces gelatinase B/MMP-9 expression in cells protected against MAC-mediated lysis by CD59 [355], suggesting that activation of the complement system may promote tumour-associated gelatinase B/MMP-9 expression. Gelatinase B/MMP-9 also degrades the β2 subunit of macrophage CD18 integrin receptor, important for macrophage recruitment [91].

A role for gelatinase B/MMP-9 has also been reported in the development of tumour tolerance. This has been attributed to gelatinase B/MMP-9 induction of tolerogenic dendritic cells (tDC), through the release and activation of TGFβ, which increases the number of regulatory T (Treg) lymphocytes that promote tumour tolerance by suppressing CD8+ cytotoxic T cells [356,357,358]. In support of this, inhibition of gelatinase B/MMP-9 expression blocks tDC development and increases tDC and Treg numbers in cancer tissues [356,358,359,360,361]. Furthermore, the VEGF/gelatinase B/MMP-9 axis promotes hyperactive haematopoiesis, expanding myeloid-derived suppressors of T-lymphocyte proliferation and activation, which results in the repression tumour immune surveillance, which promotes tumour progression [255,256,257,258,259]. 

## 17. Gelatinase B/MMP-9 Haematogenous and Lymphatic Metastatic Dispersal

Tumour cell intravasation represents an important mechanism for haematogenous metastatic dissemination. The breaching of the vascular wall is considered to represent a rate limiting step for intravasation and consequently for haematogenous metastasis [311].

**Figure 6:**
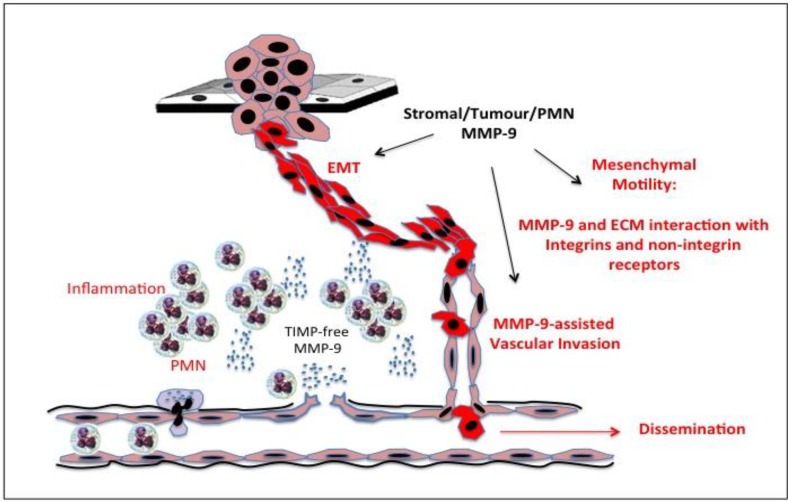
Representation of the roles played by inflammatory polymorphonuclear leukocyte (PMN)-derived tissue inhibitor of metalloproteinase (TIMP)-free gelatinase B/MMP-9, and gelatinase B/MMP-9 of stromal and tumour origin, in epithelial-mesenchymal transition (EMT) and subsequent integrin and non-integrin mediated mesenchymal motility and invasion and immature tumour blood vessels.

Gelatinase B/MMP-9 has been implicated in intravasation and subsequent metastasis formation [362] (Figure 6), with a particular role for inflammatory neutrophil-derived gelatinase B/MMP-9 highlighted in the promotion of haematogenous tumour cell dispersion of HT1080 fibrosarcoma and PC3 prostate carcinoma cells in xenograft models [12]. Neutrophil-derived gelatinase B/MMP-9 involvement in tumour-associated intravasation requires neutrophil attraction to the activated endothelial cell surface, neutrophil activation and release of TIMP-1-free gelatinase B/MMP-9. Activation of TIMP-1 free gelatinase B/MMP-9 releases angiogenic factors stored within the extracellular matrix, which promote endothelial sprouting and new vessel formation, and gelatinase B/MMP-9-assisted tumour cell intravasation and dissemination [363]. In support of this, gelatinase B/MMP-9 expression correlates with the intravasation and metastatic dissemination of HT-1080 fibrosarcoma cells, which is inhibited by the gelatinase B/MMP-9 inhibitor marimistat [362]. Furthermore, keratinocyte growth factor induces gelatinase B/MMP-9 expression and venous invasion by pancreatic cancer cells [364].

Gelatinase B/MMP-9 has also been implicated in lymphatic dissemination of colon cancer to lymph nodes. This mechanism involves gelatinase B/MMP-9 and the chemokine receptor CCR7. C-C chemokine interaction with CCR7 promotes gelatinase B/MMP-9 expression and lymphatic dissemination of colon cancer, whereas CCR7 knockdown reduces gelatinase B/MMP-9 expression lymphatic dissemination and lymph node metastases, implicating the CCR7/gelatinase B/MMP-9 axis in lymphatic metastatic dissemination of colon cancer [365]. In gastric cancer, lymphatic dissemination and lymph-node metastasis associate with increased expression of both Twist and gelatinase B/MMP-9 [233].

## 18. Gelatinase B/MMP-9 and Extravasation

After tumour cells that arrest in the microvasculature of distant organs they either extravasate or grow within vessels [366,367], adding to the debate as to whether extravasation is indeed a critical step in the metastatic process [368,369]. Due to the positive contribution made by inflammatory cells to the metastatic process, inflammatory cell-derived gelatinase B/MMP-9 may promote extravasation, as may tumour cell derived gelatinase B/MMP-9. Alternatively, endothelial cell clusters within metastatic sites may be primed to produce gelatinase B/MMP-9 by circulating VEGF through VEGF receptors [296], which may facilitate tumour cell extravasation across an already compromised vascular BM. 

## 19. Gelatinase B/MMP-9 and the Metastatic Niche

Gelatinase B/MMP-9 appears to be one of the genes required for tumor metastasis [6,9,10,12,369]. Organ specific metastatic tropism characterises the metastatic process and is a complex process that involves interaction between infiltrating cancer cells and the local environment [220]. Within the bone marrow, gelatinase B/MMP-9 regulates the recruitment and mobilization of hematopoietic stem and progenitor cells from the quiescent bone marrow niche to the proliferative niche, suggesting that gelatinase B/MMP-9 may play a similar role in cancer stem behaviour within the bone environment. In this process, gelatinase B/MMP-9 activated within the bone marrow, degrades anchorage proteins enabling haematopoietic stem cell (HSC) migration from the osteoblastic to the vascular niche, which promotes their proliferation [90]. This involves gelatinase B/MMP-9 degradation of soluble kit-ligand mobilizing factor from its membrane-associated moiety and also degradation of osteopontin, which together induce stem cell cycling and reduces anchorage to the osteoblastic niche [370]. Furthermore, osteoclasts activated within bone enhance gelatinase B/MMP-9 proteolytic activity, inducing further degradation of the endosteal-niche components osteopontin and membrane bound-stem cell factor [371]. Since cancer stem and normal stem cells share molecular machinery and cancer stem cells hijack physiological stem cell trafficking mechanisms [218], gelatinase B/MMP-9 is likely to play a similar role in stimulating the proliferation cancer stem cells that locate to the bone metastatic niche. 

Within non-bone metastatic niches, increased circulating levels of gelatinase B/MMP-9 have been shown to enhance the frequency of colon cancer metastasis to lung in a mouse model. This also associates with reduced size of metastases resulting from reduced tumour vascularisation associated with increased circulating angiostatin levels [372]. Furthermore, distant primary tumours have been shown to induce gelatinase B/MMP-9 in pre-metastatic lung endothelial cell clusters via VEGF receptor-1 signalling, pre-conditioning lungs to metastatic growth, indicating that distant tumours can aggressively determine specific metastatic sites by activating endothelial cells at secondary sites [296,373], in a process involving gelatinase B/MMP-9 expressed by endothelial cells and tumor-associated macrophages that fertilizes the soil necessary for metastatic growth [374]. Many metastatic tumours also release membrane vesicles that gain access to the circulation. Micro-vesicles shed by renal cancer stem cells contain pro-angiogenic factors, including gelatinase B/MMP-9, and promote the formation of a pre-metastatic niche, which is associated with unfavourable outcome [375]. Circulating hematopoietic CD45 and Col1a positive fibrocytes have also been shown to predispose the lung to B16/F10 metastases by recruiting Ly-6C (+) monocytes, in a chemokine and gelatinase B/MMP-9-dependent manner [376]. Furthermore, stromal derived factor (SDF)-1 interaction with the chemokine receptor CXCR4, which is essential for normal stem/progenitor cell function, promotes carcinogenesis, metastasis [377] and trans-endothelial migration of cancer cells by stimulating gelatinase B/MMP-9 secretion, disrupting basement membrane and inducing vascular permeability, promoting tumour cell extravasation. This mechanism promotes cancer stem cell homing to specific metastatic niches and in particular to the bone metastatic niche [222,378].

## 20. Gelatinase B/MMP-9, Apoptosis, Survival and the Mitochondria

Gelatinase B/MMP-9 regulates cellular survival and apoptosis [195,379]. Pro apoptotic effects have been described for gelatinase B/MMP-9 in the presence of proneurotrophins [380], in cerebellar neurons and retinal ganglion apoptosis [381,382], in hypertrophic growth plate chondrocytes [268] and in HL60 pro-myelocytic leukaemia cells [383]. Pro-survival effects of gelatinase B/MMP-9 have also been described during angiogenesis, through the release and activation of mitogens from matrix stores [211]. Gelatinase B/MMP-9 localises to mitochondria via Hsp70/Hsp60, and can disrupt mitochondrial structure, function and induce mitochondrial mtDNA damage, leading to diabetic retinal capillary cell apoptosis and gelatinase B/MMP-9 inhibition protects mitochondria from ultra-structural, functional and DNA damage [384,385,386], suggesting that gelatinase B/MMP-9 inhibitors may protect against mitochondrial apoptosis. Within the extracellular environment, gelatinase B/MMP-9 promotes neuronal apoptosis by degrading basement membrane laminin [61]. In contrast, siRNA down regulation of gelatinase B/MMP-9 expression induces apoptosis in human glioblastoma cells in association with Fas death receptor-mediated caspase 3 and caspase 8 cleavage, implicating gelatinase B/MMP-9 in protecting glioblastoma cells against Fas ligand-mediated apoptosis [387]. Methylation of the miR-211 gene up-regulates gelatinase B/MMP-9 expression in glioblastoma stem cells and increases their resistance to radiotherapy and chemotherapy-induced death [18]. The activation of α4β1 and CD44 bound gelatinase B/MMP-9 induces lyn/STAT/MCL-1 signalling and apoptosis in chronic lymphocytic leukemia cells, that depends upon gelatinase B/MMP-9 hemopexin and *O*-glycosylation domains [318,322] and in human medulloblastoma cells inhibition of gelatinase B/MMP-9 expression promotes apoptosis through β1 integrin and ERK activation [325]. In human mammary epithelial cells gelatinase B/MMP-9 expression reduces apoptosis by up regulating cell surface Her2/Neu expression [207].

## 21. Lessons from Gelatinase B/MMP-9 Knockout and Transgenics

Gelatinase B/MMP-9 knockout reduces intestinal adenoma formation and progression within the context of the APC-min mouse model, and has identified an important role for gelatinase B/MMP-9 released by inflammatory neutrophils in the formation, proliferation and progression of intestinal adenomas in cells, exhibiting compromised APC oncosoppressor function [202]. In contrast, gelatinase B/MMP-9 elimination in Myc/BclXl and RIP1-Tag2 models of pancreatic neuroendocrine carcinogenesis impairs tumour angiogenesis but promotes tumor invasion in association with a shift in inflammatory cell content to cathepsin expressing CD11b/Gr1 positive cells at the invasive front. Plasticity in tumour inflammatory infiltrates, therefore, can alter tumour-associated protease expression to compensate for gelatinase B/MMP-9 loss, helping to explain the MMP inhibitor-induced tumour progression described in human late stage tumor clinical trials [388]. Tumours, unable to grow in gelatinase B/MMP-9 knockout mice, grow readily following auto-transplantiation of normal mouse bone marrow by a mechanism independent of endothelial cell progenitors but involving CD11b positive myelomonocytic cells. In this model gelatinase B/MMP-9 is required for tumor-associated vasculogenesis [269]. Human pancreatic cancer cell growth, impaired in gelatinase B/MMP-9 knockout mice, is promoted by gelatinase B/MMP-9 produced by parabiosed normal stromal cells, implicating stromal gelatinase B/MMP-9 in tumour progression [272]. Gelatinase B/MMP-9 knockout mice also exhibit substantial inhibition of spontaneous metastasis due to impaired triggering of the “angiogenic switch” [389], and in experimental metastasis models, lung metastasis formation by both melanoma and lung carcinoma cells is reduced [390,391]. The inhibition of skin and ovarian cancer metastasis formation in gelatinase B/MMP-9 knockout mice can be reversed by transplantation of normal bone marrow cells, implicating inflammatory cell gelatinase B/MMP-9 in the metastatic process and adding to the role of gelatinase B/MMP-9 in primary tumour initiation, promotion and expansion [170,390].

In contrast to these reports, transgenic α1 integrin mice exhibit increased gelatinase B/MMP-9 expression and produce high level of circulating angiostatin, which reduces primary and metastatic growth of orthotopic cancers, in association with reduced angiogenesis. This identifies an anti-angiogenic, tumour suppressing function for gelatinase B/MMP-9 [392,393,394].

In mice transgenic for the gelatinase B/MMP-9 inhibitor TIMP-1, paradoxical effects have been described, with high circulating TIMP-1 inhibiting DMBA-induced mammary tumour growth, blocking tumorigenesis at an early stage [395]. In contrast, high circulating TIMP-1 promotes subcutaneous B16 melanoma growth in association with increased angiogenesis, whilst suppressing metastatic lung colonisation [396]. High circulating TIMP-1 levels, furthermore, strongly promote liver fibrosis [397], implicating gelatinase B/MMP-9 in normal liver physiology, adding to its physiological roles in the nervous system, inflammation and immunology [12,13,398].

## 22. Gelatinase B/MMP-9 Inhibitors and Future Directions

More than 50 broad-spectrum MMP inhibitors have been subjected to clinical trials. However, despite impressive results in non-randomized clinical trials, phase II and III clinical trials in patients with a range of different cancers have not been positive, due to a combination of factors that include a lack of understanding of the complexities of MMP involvement in tumour pathogenesis and progression, the lack of inhibitor specificity, drug intolerance and problems with drug dosage [399,400,401]. This was somewhat expected considering reports that several MMPs, including gelatinase B/MMP-9, display anti-tumor activity [72,112,113,284,285,402,403], different MMPs may be involved in different stages of tumour progression and the most potent endogenous gelatinase B/MMP-9 inhibitor, TIMP-1 may promote scattered micro-metastases in the liver [404]. Therefore, the detailed characterisation of exact roles played by the different MMPs within tumour pathogenesis and progression is required, as is the development of highly specific MMP inhibitors.

The impressive quantity of data concerning gelatinase B/MMP-9 involvement in the different phases of tumor progression, reviewed in this article, highlights particularly important roles for inflammatory leukocyte-derived gelatinase B/MMP-9 in tumour initiation and early progression and a more complex involvement of gelatinase B/MMP-9 from inflammatory, stromal and tumour sources in the continued progression of tumours to metastasis. Furthermore, reports also suggest that under certain conditions gelatinase B/MMP-9 may also protect against tumour progression by promoting the formation of systemic inhibitors of angiogenesis, may promote apoptosis and also facilitate anti-tumor inflammatory and immunological reactions (see Section 11 and Section 16). Furthermore, it is also evident that under conditions of MMP inhibition malignant tumour cells compensate by undergoing mesenchymal to amoeboid transition, facilitating protease-independent progression [405].

Current gelatinase B/MMP-9 inhibitors can be divided into those that inhibit gelatinase B/MMP-9 expression or catalytic activity. NSAIDs and HMG-CoA reductase inhibitors inhibit gelatinase B/MMP-9 transcription [238]. Gelatinase B/MMP-9 inhibitory siRNA inhibits gelatinase B/MMP-9 expression and tumorigenicity in a model of medulloblastoma [325] and miR-491-5p, miR-885-5p and miR-211 inhibit gelatinase B/MMP-9 expression and involvement in models of human glioblastoma [17,18]. Gelatinase B/MMP-9 activity can be blocked by the broad range MMP inhibitors d-penicillamine, hydroxamates, bisphosphonates and tetracyclins, [406]. An active site-specific gelatinase B/MMP-9 inhibitory antibody REGA-3G12 has been reported [407], and bisphosphonates inhibit both gelatinase B/MMP-9 expression and activity [41,255]. Zoledronic acid has been shown to inhibit macrophage gelatinase B/MMP-9 and reduces angiogenesis in a model of papillomavirus-induced cervical cancer [255]. Alendronate inhibits gelatinase B/MMP-9 activity and promotes plasmin-mediated destruction of the gelatinase B/MMP-9 catalytic domain, promoting irreversible gelatinase B/MMP-9 inhibition and producing inhibitory gelatinase B/MMP-9 hemopexin fragments, suggesting a novel rational for Alendronate use in pathology dependent upon gelatinase B/MMP-9 activity and plasminogen activation [41].

However, with the exception of REGA-3G12, there are few specific inhibitors of gelatinase B/MMP-9 catalytic activity, attesting to the close similarity exhibited by MMP catalytic sites. A HFDDDE motif of the gelatinase B/MMP-9 catalytic domain interferes with pro-gelatinase B/MMP-9 binding of β2 integrin, inhibits OCI-AML3 leukemia cells transmigration across a human endothelial cell layers and inhibits gelatinase B/MMP-9-mediated extracellular matrix degradation, suggesting potential use in therapeutic reduction of acute myeloid leukemia cells extra medullary infiltration [408]. The peptides CTTHWGFTLC and GRENYHGCTTHWGHTLC also inhibit gelatinase B/MMP-9 catalytic activity but not gelatinase B/MMP-9 activation and inhibit primary HSC-3 tongue carcinoma growth but not tumor spread in a mouse model [409].

Recent approaches have also focussed on molecules that interfere with gelatinase B/MMP-9 activity mediated by the hemopexin and/or O glycosylation domains. Recombinant or isolated gelatinase B/MMP-9 hemopexin domain inhibits gelatinase B/MMP-9 activity [41,163], hampers colorectal cancer cell adhesion and migration [410], inhibits gelatinase B/MMP-9-induced functions in chronic lymphocytic Leukemia B cells [411], and inhibits angiogenesis in glioblastoma xenografts [291]. A peptide mimic of integrin beta chain that binds the gelatinase B/MMP-9 hemopexin domain inhibits gelatinase B/MMP-9 binding to αVβ5 integrin, prevents progelatinase B/MMP-9 activation, inhibits HT-1080 fibrosarcoma cell invasion *in vitro* and HSC-3 tongue carcinoma xenograft growth *in vivo* but does not inhibit gelatinase B/MMP-9 activity [412]. Peptide mimics of the first and fourth blades of the gelatinase B/MMP-9 hemopexin domain block gelatinase B/MMP-9 dimerization and inhibit HT-1080 and MDA-MB-435 tumour cell motility [48]. The compound *N*-[4-(difluoromethoxy) phenyl]-2-[(4-oxo-6-propyl-1H-pyrimidin-2-yl) sulfanyl]-acetamide also binds to the gelatinase B/MMP-9 hemopexin domain, inhibits gelatinase B/MMP-9 homo-dimerization, blocks gelatinase B/MMP-9 mediated migration and reduces xenograft tumorigenicity and metastasis of MDA-MB-435 human breast cancer cells [46]. Deletion of the *O*-glycosylation domain inhibits macromolecular substrate specificity of gelatinase B/MMP-9 [413], suggesting that inhibitors of *O*-glycosylation domain function may also be effective inhibitors of gelatinase B/MMP-9 function. Therefore, molecules that interact, interfere or compete with these two domains hold some promise in the development of specific therapeutic inhibitors of gelatinase B/MMP-9 activity and function. When considering the potential therapeutic use of specific gelatinase B/MMP-9 inhibitors, however, potential anti-tumor activity of gelatinase B/MMP-9 (*i.e.*, production of anti-angiogenic molecules) must be taken into account and attempts made also to limit inhibitor interference with physiological gelatinase B/MMP-9 functions.

## 23. Conclusions

There is no doubt that gelatinase B/MMP-9 plays a fundamental role in tumour biology, ranging from initiation/promotion to angiogenesis, dissemination, immunological surveillance and metastatic growth. Gelatinase B/MMP-9, however, also exhibits anti-tumor activity and plays important physiological functions. It is therefore essential that specific inhibitors of gelatinase B/MMP-9 proteolytic and non-proteolytic functions are developed in order to determine the potential therapeutic efficacy of inhibiting gelatinase B/MMP-9 function in cancer therapy. The difficulty will be to inhibit the tumour promoting functions of gelatinase B/MMP-9, whilst substituting for anti-tumor gelatinase B/MMP-9 effects and minimising the inhibition of physiological gelatinase B/MMP-9 function.
